# Biocatalytic Degradation Efficiency of Postconsumer Polyethylene Terephthalate Packaging Determined by Their Polymer Microstructures

**DOI:** 10.1002/advs.201900491

**Published:** 2019-05-20

**Authors:** Ren Wei, Daniel Breite, Chen Song, Daniel Gräsing, Tina Ploss, Patrick Hille, Ruth Schwerdtfeger, Jörg Matysik, Agnes Schulze, Wolfgang Zimmermann

**Affiliations:** ^1^ Department of Microbiology and Bioprocess Technology Institute of Biochemistry Leipzig University Johannisallee 23 D‐04103 Leipzig Germany; ^2^ Leibniz Institute of Surface Engineering (IOM) Permoserstrasse 15 D‐04318 Leipzig Germany; ^3^ Institute of Analytical Chemistry Leipzig University Linnéstrasse 3 D‐04103 Leipzig Germany; ^4^ AB Enzymes GmbH Feldbergstrasse 78 D‐64293 Darmstadt Germany

**Keywords:** crystallinity, enzymatic degradation, physical aging, polyethylene terephthalate, postconsumer packaging

## Abstract

Polyethylene terephthalate (PET) is the most important mass‐produced thermoplastic polyester used as a packaging material. Recently, thermophilic polyester hydrolases such as TfCut2 from *Thermobifida fusca* have emerged as promising biocatalysts for an eco‐friendly PET recycling process. In this study, postconsumer PET food packaging containers are treated with TfCut2 and show weight losses of more than 50% after 96 h of incubation at 70 °C. Differential scanning calorimetry analysis indicates that the high linear degradation rates observed in the first 72 h of incubation is due to the high hydrolysis susceptibility of the mobile amorphous fraction (MAF) of PET. The physical aging process of PET occurring at 70 °C is shown to gradually convert MAF to polymer microstructures with limited accessibility to enzymatic hydrolysis. Analysis of the chain‐length distribution of degraded PET by nuclear magnetic resonance spectroscopy reveals that MAF is rapidly hydrolyzed via a combinatorial exo‐ and endo‐type degradation mechanism whereas the remaining PET microstructures are slowly degraded only by endo‐type chain scission causing no detectable weight loss. Hence, efficient thermostable biocatalysts are required to overcome the competitive physical aging process for the complete degradation of postconsumer PET materials close to the glass transition temperature of PET.

## 1 Introduction

The global annual production of plastics has been estimated to reach 348 million tons (Mt.) in 2017,^1^ of which 90% was produced from fossil fuels.^2^ Packaging is the largest application sector making up close to 40% of the total plastics demand.^1^ The short‐lived, disposable packaging materials greatly contribute to the municipal solid waste and marine anthropogenic litter, which have caused a serious worldwide environmental crisis.^3^ Recycling of postconsumer plastics by recovering its monomers as raw materials for new polymer production has been suggested as the most economic approach to treat plastic waste, and thereby closing the loop of a circular economy.^1^, ^4^

Polyethylene terephthalate (PET) is a semicrystalline thermoplastic polyester and is mainly processed to beverage bottles and other containers used for food packaging.^1^, ^5^ The global annual production of PET resins is ≈33 Mt. while a similar amount of PET waste is generated every year mainly composed of postconsumer packaging materials.^6^ Recently, a biological recycling of PET to regain its monomers terephthalic acid and ethylene glycol has been suggested as an alternative to the currently performed mechanical and chemical recycling processes.[qv: 3c,7] PET shows a high recalcitrance to biological degradation although its monomers are connected by simple ester bonds.^8^ Previously identified PET‐hydrolyzing enzymes were found within several small homologous groups.[qv: 7b,9] The report of a mesophilic PET‐assimilating bacterium *Ideonella sakaiensis* has recently attracted the attention of the scientific and general audience.^10^ However, the PET‐hydrolyzing enzyme of this strain with a temperature optimum of 40 °C was shown to be markedly less efficient in the PET degradation compared to selected thermophilic counterparts.[qv: 7b,]10–11 This is due to a high glass transition temperature of PET of around 75 °C where the amorphous PET domains gain enough mobility to be readily accessed by the enzyme.[qv: 7b,11] It has also been shown that the crystallinity of a PET sample strongly influences its degradation efficiency catalyzed by various polyester hydrolases.^10^, ^11^, ^12^ These previous studies indicated that the high degree of crystallinity above 20% of PET bottles and fibers was correlated with a considerably low enzymatic degradation rate. Although a pretreatment at high temperature (250 °C) and pressure (39 bars) could convert highly crystalline PET fibers into biodegradable oligomers,^13^ this process was much more energy consuming than an enzymatic hydrolysis of postconsumer PET materials.

Many previous studies assessed only the degree of crystallinity after an enzymatic PET degradation and compared with an untreated control while the change of the polymer properties during the enzymatic hydrolysis has remained unexplained. In this study, we used a thermostable recombinant polyester hydrolase expressed in *Bacillus subtilis* for the degradation of postconsumer PET food packaging containers. Untreated and partially hydrolyzed polymer samples were analyzed by differential scanning calorimetry, Fourier‐transform infrared spectroscopy, nuclear magnetic resonance spectroscopy, and scanning electron microscopy. The extent of the enzymatic degradation of the different amorphous and crystalline microstructures of the polymer as well as the effects of a physical aging process of the PET polymer during incubation with the enzyme at 70 °C was evaluated.

## 2 Results and Discussion

### 2.1 *B. subtilis* Expressed a More Active and Thermostable TfCut2 Compared to *Escherichia coli*

**Figure 1:**
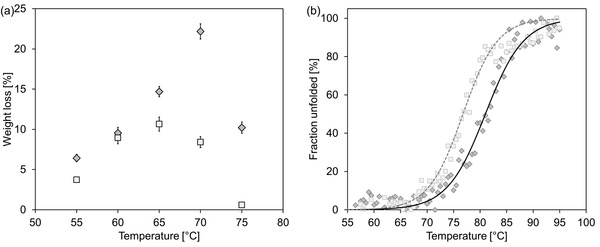
Comparison of recombinant TfCut2 expressed in *B. subtilis* (diamonds) and *E. coli* (squares). a) Averaged percentage of weight loss of GF‐PET chips of 0.5 × 3 cm^2^ after incubation at various temperatures for 24 h. Error bars indicate the standard deviation of triplicate determinations. b) Thermal denaturation curve of TfCut2 determined by CD spectroscopy. The fitting curves for the determination of the melting points are shown as a black solid curve and as a grey dashed curve for TfCut2 expressed in *B. subtilis* and *E. coli*, respectively.

Recombinant *Thermobifida fusca* cutinase TfCut2 was overexpressed in *B. subtilis* and obtained as the only dominant protein in the cell‐free culture supernatant after 42 h of cultivation at 37 °C (Figure S1a, Supporting Information). Compared to the previously reported expression of a highly similar enzyme TfH (99% sequence identity to TfCut2) in *Bacillus megaterium*,^14^ TfCut2 could be obtained in higher purity in the growth medium of *B. subtilis* thereby enabling a straightforward purification of the recombinant enzyme by a single size exclusion chromatography step to yield a highly pure enzyme preparation (Figure S1c, Supporting Information). When amorphous GF‐PET (Goodfellow Ltd., Bad Nauheim, Germany) chips were hydrolyzed with TfCut2 at various temperatures for 24 h, the degradation performance was shown to be dependent on the expression hosts used (**Figure** **1**a). A weight loss of 22.2 ± 0.9% of the GF‐PET sample could be achieved at 70 °C with TfCut2 expressed in *B. subtilis*, while the *E. coli*‐derived enzyme caused a maximal weight loss of 10.6 ± 0.9% at 65 °C, similarly as reported previously.^15^ As shown in Figure 1b, the apparent melting points of TfCut2 expressed in *B. subtilis* and *E. coli* determined by circular dichroism (CD) spectroscopy were 80.7 ± 0.3 °C and 76.6 ± 0.3 °C, respectively, suggesting a more stable overall structure and superior thermostability of the former. Considering the extracellular nature of TfCut2, the secretory expression using *B. subtilis* was regarded as a more favorable condition for its stable folding compared to the reducing intracellular milieu in *E. coli*.^16^

### 2.2 Different Enzymatic Degradability of Amorphous PET Film and PET Postconsumer Packages

In this study, the weight loss of bulky PET materials after enzymatic hydrolysis was determined to evaluate the degradation performance. These values indicated the depolymerization of the PET polymers into monomers such as terephthalic acid (TPA) and ethylene glycol (EG) as well as their mono‐ and di‐esters, the release of which could be easily monitored by reversed phased high performance liquid chromatography (HPLC) and correlated with the weight loss determined with the same sample.^17^ Enzymatic and chemical hydrolysis of PET at neutral and alkaline condition result in a comparable composition of the soluble degradation products, from which EG can be easily recovered by distillation and TPA by filtration following its precipitation in the presence of a strong mineral acid.^18^

**Figure 2:**
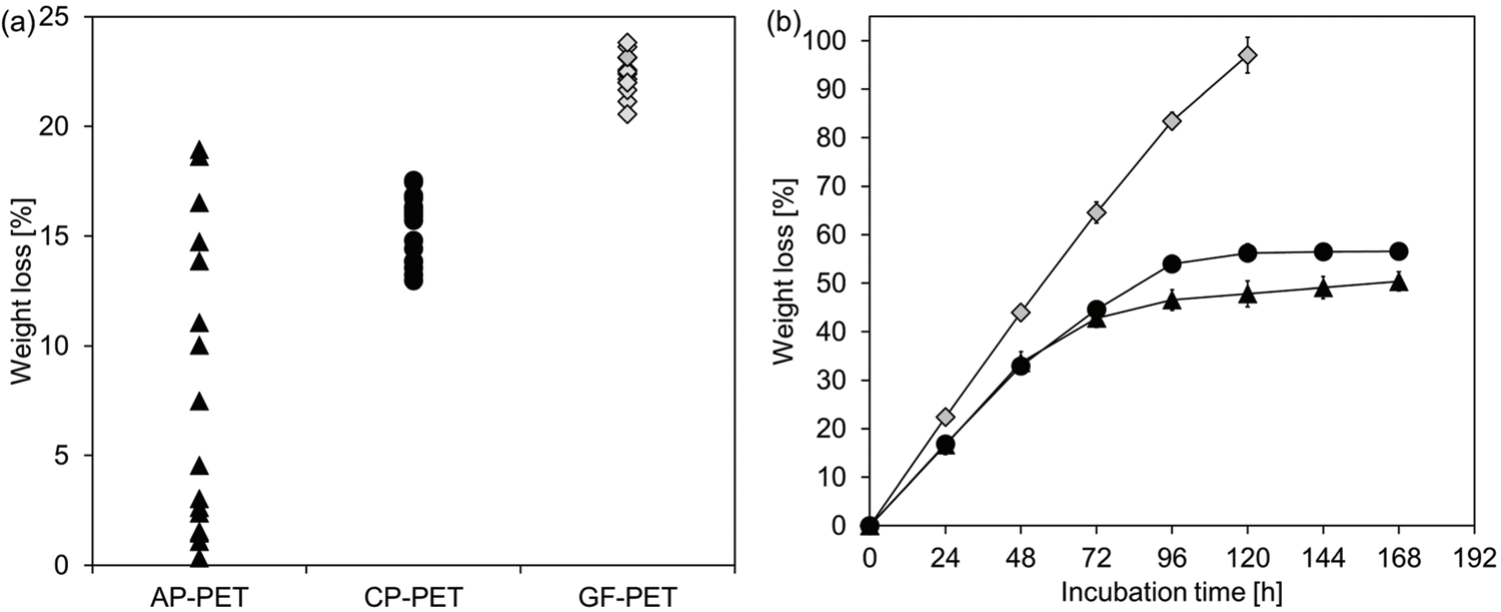
Weight loss of PET chips from AP‐PET (triangles), CP‐PET (filled circles), and GF‐PET (diamonds) hydrolyzed by TfCut2. a) Weight loss of up to 15 chips sampled from different parts of the PET samples after 24 h of incubation with the enzyme at 70 °C. b) Degradation of selected PET chips which showed the highest weight loss in (a) determined over an incubation time of 168 h with the enzyme. Error bars indicate the standard deviations obtained from at least triplicate experiments.

As shown in **Figure** **2** (Figure S2a, Supporting Information), PET samples from postconsumer packages (AP‐PET, Agripack, Groupe Guillin, Ornas, France; CP‐PET, Carton Pack Srl, Rutigliano, Italy) and from amorphous film (GF‐PET) hydrolyzed by the *B. subtilis* expressed TfCut2 at 70 °C revealed different degradation performance. In the first 24 h of degradation, weight losses of CP‐PET (15.3 ± 1.5%) and GF‐PET (22.3 ± 0.9%) chips sampled from different parts of the packages and films were detected corresponding to absolute weight losses of 4.8 ± 0.5 mg and 9.9 ± 0.1 mg, respectively. In contrast, AP‐PET chips showed a different biodegradability depending on the origin of the chips sampled from different parts of the packages. Weight losses of the chips were detected in a broad range from 0.3% to 18.9%, corresponding to a maximum absolute weight loss of 7.3 mg (Figure 2a). With the same enzyme concentration per surface area of the chips (1 nmol cm^−2^) applied to all PET samples, the amorphous GF‐PET showed the highest biodegradability.

**Figure 3:**
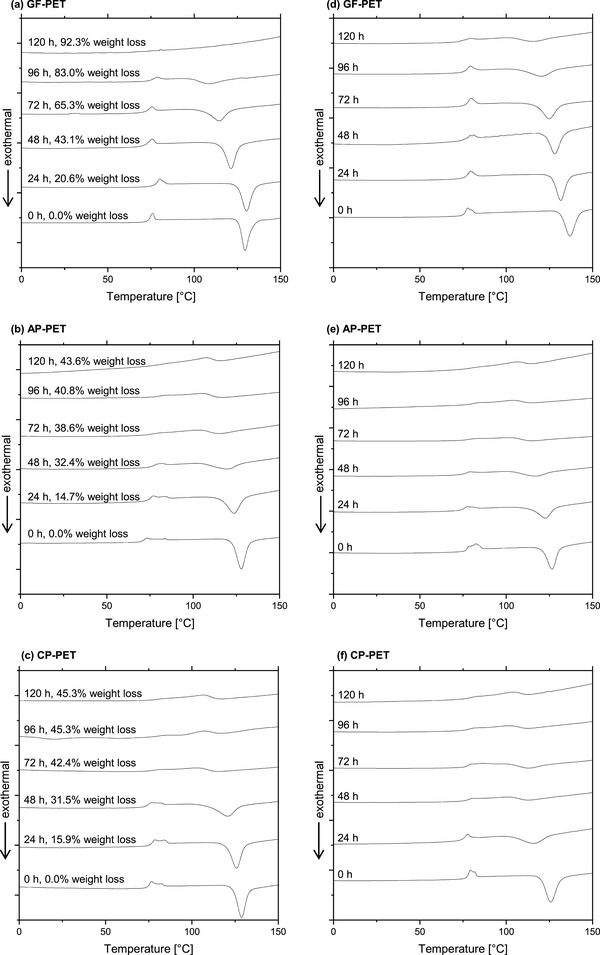
First DSC heating scans of the PET samples. a–c) GF‐PET, AP‐PET, and CP‐PET samples treated with TfCut2 at 70 °C for different time periods and d–f) samples incubated in buffer only.

The time course of the enzymatic degradation during a reaction time of 168 h at 70 °C is shown in Figure 2b with PET chips removed from the most hydrolysis‐susceptible packaging parts. The amorphous GF‐PET chips were almost completely degraded (97.0 ± 3.0%) within 120 h at a linear rate of 20–22% per day in the first 96 h. These rates were slightly lower than those obtained previously with a fungal cutinase (HiC) at 70 °C.^11^ From ten individual GF‐PET chip samples, only one left recoverable bulky materials which were characterized later by differential scanning calorimetry (DSC) (**Figure** **3**a). In contrast to the amorphous GF‐PET film, the most hydrolysis‐susceptible PET chips from the packages showed a lower linear degradation rate of up to 17% per day up to 48 h of reaction. Subsequently, the degradation curves leveled off to almost no further degradation observed during longer reaction times from 120 to 168 h. AP‐ and CP‐PET chips from different parts of the postconsumer packages showed a strong discrepancy in their degradability after 24 h incubation, as indicated by wide ranges covered by the degradation curves (Figure S2b,c, Supporting Information). After 168 h of incubation, TfCut2 resulted in maximum weight losses of 50.5 ± 1.2% and 56.6 ± 1.4% with AP‐ and CP‐PET chips, respectively. At the same condition, minimum weight losses of 8.2 ± 1.2% for AP‐PET and 23.9 ± 2.4% for CP‐PET were also determined (Figure S2b,c, Supporting Information). These results suggest a heterogeneous distribution of amorphous and crystalline microstructures correlating with different enzymatic degradability within the PET samples. The heterogeneity of the different transparent PET packaging samples is likely the result of their thermoforming process which resulted in local strain‐induced polymer crystallization and discontinuous microstructures.^19^ While crystals smaller than the wavelength of the visible light will not reduce the transparency of the PET material, they will still restrict the mobility of the amorphous polymer chains in their neighborhood thereby affecting its overall biodegradability.^20^

### 2.3 Changes of the Microstructure of the PET Samples During Enzymatic Degradation

DSC analysis of selected PET samples incubated with TfCut2 and with only buffer for up to 120 h is shown in Figure 3. The first heating scans of the untreated GF‐PET (*t* = 0 h) revealed a glass transition temperature (*T*_g_) between 75 and 79 °C overlayed by an endothermic signal and a cold crystallization temperature (*T*_cc_) between 130 and 138 °C as indicated by an exothermic peak in this temperature range (Figure 3a,d). *T*_g_ and *T*_cc_ values of PET determined by DSC are dependent on the heating rate performed.^21^ Using the same heating rate of 10 °C min^−1^ by DSC, the *T*_g_ obtained in this study were in good agreement with a previous study using the same amorphous PET material with however a significant higher *T*_cc_ of 142 °C.^11^ The cold crystallization temperature range is dependent on the magnitude of the nucleation and the rates of crystal growth.^21^ Therefore, the GF‐PET used in this study appeared to be more prone to crystallization than the material used by Ronkvist et al. (2009) which demanded higher temperatures for initiating the cold crystallization.^11^ In comparison with GF‐PET, the postconsumer PET packaging materials revealed two separate endothermic peaks in the glass transition range, corresponding to a *T*_g1_ of 75–78 °C and a *T*_g2_ of 83–85 °C for AP‐PET and a *T*_g1_ of 77–80 °C and a *T*_g2_ of 83–85 °C for CP‐PET (Figure 2b,c,e,f). Only one of the untreated GF‐PET samples (*t* = 0 h) among more than ten samples also showed a *T*_g1_ of 78 °C and a *T*_g2_ of 80 °C (Figure 3d). In a low‐crystalline PET sample, the existence of dual amorphous phases with different conformational mobility has been shown to depend on its initial crystallinity and previous thermal history.[qv: 20a,b,22] A mobile amorphous fraction (MAF, *T*_g1_) has a chain mobility similar to the purely amorphous polymer whereas a rigid amorphous fraction (RAF, *T*_g2_) shows a lower chain mobility due to its presence within the intraspherulitic or interlamellar regions in close vicinity to the crystalline domains.[qv: 20b,22a] The presence of RAF in semicrystalline aromatic polyesters has been described by Menczel and Wunderlich in the 1980s.^23^ The RAF present in both postconsumer PET samples and their higher *T*_g2_ than GF‐PET suggested that the latter has less ordered microstructures. This can be further verified by the lower *T*_cc_ of postconsumer PET samples between 126 °C and 130 °C determined compared to GF‐PET which is more amorphous and thus requires higher temperatures to initiate the cold crystallization.^21^ The melting temperatures of the three types of PET materials were all in the range of 245–251 °C (results not shown) in agreement with previous studies.[qv: 11,12c,21,24] At longer incubation times up to 120 h at 70 °C, a shift of the *T*_g_ to higher values and the disappearance of the lower glass transition peaks was observed after treatment of the PET samples both in the presence and absence of the enzyme (Figure 3a–c). This result can be attributed to the transition from the MAF to the RAF in the amorphous microstructures of PET resulting in a lower total chain mobility.[qv: 20a] Concomitantly, a shift of *T*_cc_ to a lower temperature range and a decreasing intensity of the cold crystallization peak was also observed in all PET samples. The exothermal cold crystallization occurs spontaneously when a part of the amorphous PET crystallizes. Therefore, the decreasing intensity of the cold crystallization peaks suggested the shrinkage of the amorphous microstructures in the PET samples which were in a thermodynamic nonequilibrium state.[qv: 20a,25] Incubation of the amorphous PET samples at 70 °C close to the *T*_g_ of PET caused their local molecular architectures to rearrange toward a higher order resulting in a state closer to equilibrium.[qv: 25a] This process is generally termed as structural relaxation or physical aging.[qv: 20a,25] Indeed, no significant differences were observed between the DSC thermograms obtained with PET samples enzymatically degraded or those undergone physical aging when incubated with buffer only. These results suggest that the property changes of the residual bulk PET polymers during enzymatic degradation were mainly caused by the physical aging process rather than by the enzymatic degradation.

**Figure 4:**
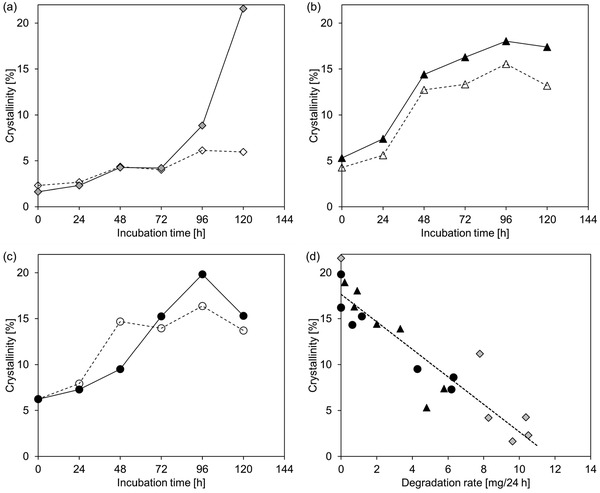
Change of the initial crystallinity of the PET samples due to enzymatic degradation and the accompanying physical aging at 70 °C. a–c) Crystallinity determined by DSC of a single PET chip of GF‐PET (filled diamonds), AP‐PET (filled triangles), and CP‐PET (filled circles) as a function of the incubation time. Empty symbols and dashed lines indicate data obtained with samples incubated without enzyme. d) Relationship between the crystallinity of single PET chips and their degradation rates (mg weight loss in 24 h). A linear fitting (dashed line) with a regression coefficient of higher than 0.92 is shown.

**Figure 5:**
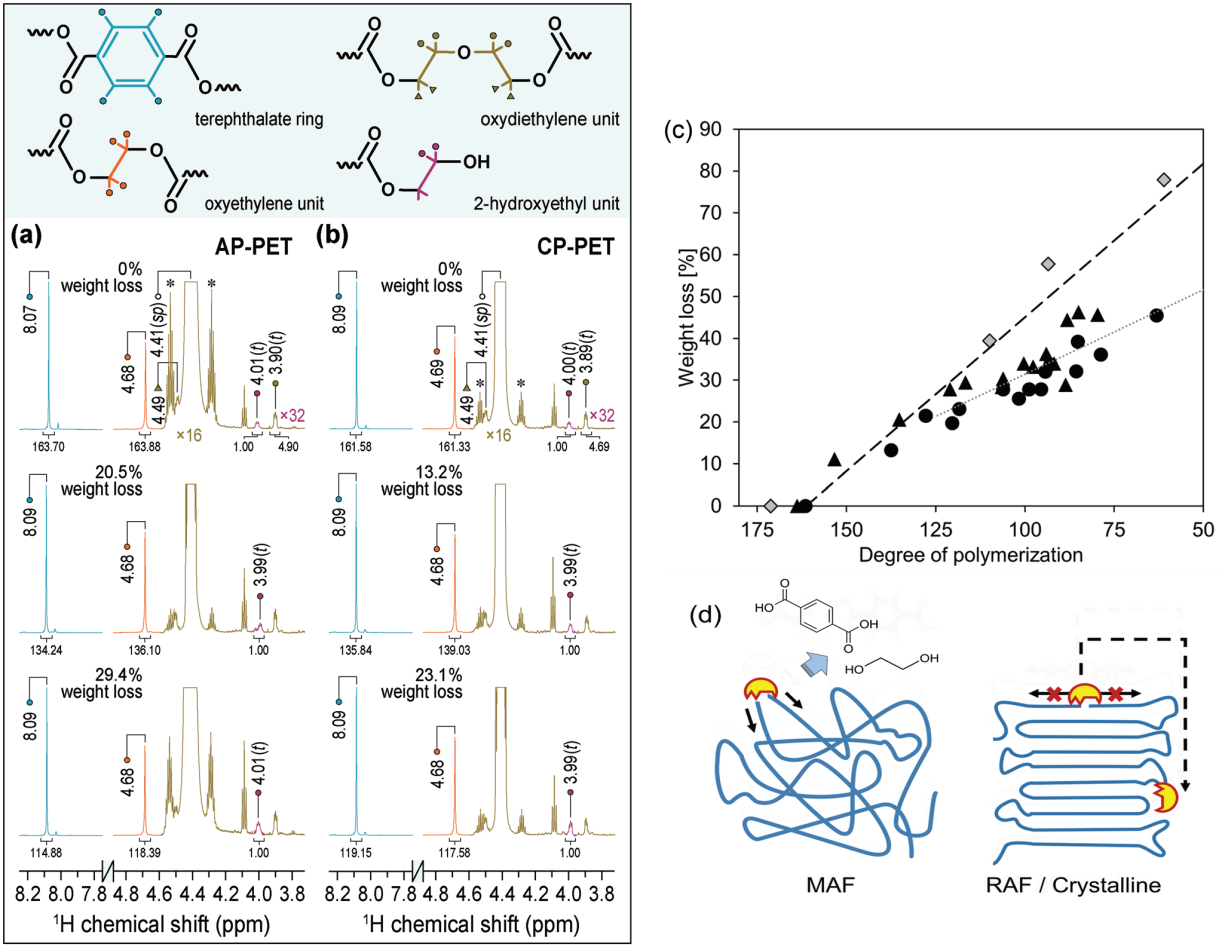
NMR analysis of the PET samples hydrolyzed by TfCut2 suggesting the degradation mechanisms of different PET microstructures. ^1^H spectra of a) AP‐PET and b) CP‐PET recorded in deuterated CDCl_3_/HFIP (16:1, v/v): only spectral regions of 7.8–8.2 ppm and 3.8–4.8 ppm are shown. Integration ratios are given under each signal calculated relative to the CH_2_ signal at around 4.0 ppm. To obtain better visibility, intensities of ^1^H signals in yellow and purple are increased by a factor of 16 and 32, respectively. The ^13^C satellites of the parent HFIP signal (δ^H^, 4.41 ppm) are denoted by asterisks. c) The relative weight loss of the samples is plotted as a function of the DP of GF‐PET (diamonds), AP‐PET (triangles), and CP‐PET (filled circles), with the fitting line (dashed) based on GF‐PET, AP‐PET, and CP‐PET samples with less than 30% weight loss and a fitting line (dotted) based on other AP‐PET and CP‐PET samples with higher weight loss. d) A schematic illustration of the hypothesized enzymatic degradation mechanisms of different PET microstructures. Chemical structures of ethylene glycol and terephthalic acid are illustrated as the degradation products of PET indicating the exo‐type chain scission.

Based on the cold crystallization enthalpy and the melting enthalpy values, the change of the degree of crystallinity of the PET samples during the incubation with the enzyme or buffer only was calculated according to Equation (1) (**Figure** **4**). Untreated AP‐ and CP‐PET with an initial degree of crystallinity in the range of 4–6% can be thus categorized as amorphous PET packaging materials, which were produced using precursor amorphous materials like the GF‐PET films by thermoforming process at a low temperature of around 90 °C.^26^ The time courses showed that the crystallinity of all PET samples increased during the incubation at 70 °C. The crystallinity of the GF‐PET incubated without enzyme increased from 2.3% to about 6% over 120 h as a result of physical aging of the material at 70 °C (Figure 4a). In contrast, when incubated without enzyme, the AP‐PET and CP‐PET samples showed a steeper increase in their crystallinity from 4.3% to more than 13% (Figure 4b) and from 6.2% to more than 14% (Figure 4c), respectively, during the first 48 h of incubation. At longer incubation times, the crystallinity increased more slowly indicating that an apparent equilibrium of the molecular rearrangement of the polymer chains characterized by the transition from MAF to RAF was almost reached after 48 h.[qv: 20a] Concomitantly, significantly lower rates of the apparent weight losses of the AP‐PET and CP‐PET samples degraded by TfCut2 were observed after 48 h of incubation (Figure 2b), suggesting that the RAF with a markedly lower chain mobility could not be hydrolyzed by the enzyme. The observed physical aging of the PET materials at 70 °C can therefore be considered as competitive to the enzymatic degradation of the amorphous PET. The untreated GF‐PET sample with a very low crystallinity of 2.3% and without an apparent thermal pretreatment history can be expected to contain a high proportion of MAF which was degraded at a nearly constant high rate within 120 h of incubation (Figure 2b). Although a moderate increase in crystallinity resulting from physical aging was also observed with the GF‐PET, a significantly lower degradation rate as a result of the transition from MAF to RAF was not observed, implying that the MAF is indeed the hydrolysis‐susceptible part of the amorphous microstructures of PET. Most of the GF‐PET samples were completely degraded after 120 h of incubation and only one residual sample showing an increase of the crystallinity to 21.6% (Figure 4a) was obtained and cannot be further enzymatically degraded. This result is consistent with a previous study which has reported a crystallinity of 27% determined with a residual amorphous PET sample after incubation for 96 h at 70 °C with the polyester hydrolase HiC.^11^ Furthermore, an increase of the crystallinity by 2% as a result of physical aging of the amorphous PET at 70 °C incubated without enzyme has also been observed in their study. An increase in crystallinity of PET samples by 2–3% as a result of physical aging at 60 °C for 72 h, and by 6–8% following incubation with a polyester hydrolase has also been reported.[qv: 12d] The lower increase of crystallinity of the PET observed in their study could be explained by the fact that the time required to reach a thermodynamic equilibrium during the physical aging process is dependent on the incubation temperature.[qv: 20a] The observed increase of the crystallinity of the amorphous PET samples can be attributed to a synergistic effect of both physical aging and the preferential degradation of the MAF microstructure of the PET polymer. In comparison, the postconsumer PET samples with a significantly higher content of RAF showed a larger increase of their crystallinity of up to 15% due to the physical aging process (Figure 4b,c). The crystallinity of the samples incubated with the polyester hydrolase TfCut2 increased further up to 20% after 96 h of incubation, although almost no further weight loss could be detected after 72 h (Figure 2b). This could be explained by an internal reduction of the length of the PET polymer chains by the enzyme as confirmed by nuclear magnetic resonance (NMR) analysis without a release of the breakdown products (see **Figure** **5**c below). The shorter PET polymer chains could have fold among themselves and rearranged into intercrystalline domains resulting in a higher crystallinity compared to the physically aged samples.^27^

The crystallinity of PET samples showed an inverse linear relationship to the observed weight loss rates (Figure 4d). Above a crystallinity of 20%, weight losses were not detected since the RAF domains with constrained chain mobility were dominant in the PET samples.[qv: 20a] This observation further suggests that only the more flexible MAF of PET are accessible to the polyester hydrolases and not the total amorphous microstructures. Several previous studies have pointed out the relationship of the biodegradability and the initial crystallinity of PET.^10^, ^11^, ^12^ In agreement with these reports our results showed that an enzymatic degradation of PET with higher than 20% crystallinity did not result in any apparent weight loss of the material.[qv: 12e]

### 2.4 NMR Analysis Confirmed Endo‐ and Exo‐type Scissions of the PET Polymer Chains by TfCut2

The NMR spectra of the postconsumer PET samples AP‐PET and CP‐PET at different stages of enzymatic degradation are shown in Figure 5a,b. Both spectra of the untreated PET samples revealed two sharp singlets of nearly equal NMR integrals (<± 1%) at around 8.1 and 4.7 ppm, corresponding to four protons from the terephthalate ring and the oxyethylene units in the main chain, respectively.^28^ These two signals did not exhibit a detectable ^1^H shift in the spectra of the CP‐PET during enzymatic degradation (Figure 5b). In comparison, the AP‐PET showed a 0.02 ppm downfield shift of the ^1^H signal for terephthalate protons compared to their pristine counterpart before enzymatic degradation. Two triplets at δ^H^ of 3.90 (2H, *t*, ^3^ *J* = 5.8 Hz) and 4.49 (2H, *t*, ^3^ *J* = 5.8 Hz) ppm were assigned to the protons of the two oxydiethylenic methylenes.[qv: 28a] Diethylene glycol can form during the PET synthesis, and enters subsequently into the growing polyester chains resulting in the formation of an oxydiethylene unit.[qv: 28b] The corresponding chain defects lower the glass transition temperature of PET and the extent of its crystalline microstructures.^29^ By comparing the ^1^H integrals for oxydiethylene and terephthalate units, mole fraction of the former of ≈2.99% and 2.91% were calculated for the AP‐PET and CP‐PET samples, respectively. Consistently, previous studies have shown oxydiethylene units in the same fraction value ranges in other commercial PET samples.^28^

A relatively weak but well‐resolved ^1^H signal appearing as a triplet at δ^H^ of 4.0 ppm (2H, *t*, ^3^ *J* = 4.7 Hz, colored in purple in Figure 5a,b) was assigned to the methylene protons α adjacent to the hydroxyl end group of the PET chains.[qv: 28a] By comparing the ^1^H integrals for the hydroxyl end groups and the terephthalate units, a mole fraction of 0.61% and 0.60% was determined in the untreated AP‐PET and CP‐PET, respectively. Assuming that each PET polymer chain has only one hydroxyl end group, an apparent degree of polymerization (DP) of 161.7 and 166.7 was obtained for AP‐PET and CP‐PET, respectively, corresponding to an apparent number average molecular weight ($\bar{M}$_n_) of 31 500 and 31 000 g mol^−1^, respectively, by applying the molecular weight of 192.2 g mol^−1^ of a single PET repeating unit. These values are in the same order of magnitude of $\bar{M}$_n_ of PET samples determined previously by size exclusion chromatography.^10^, ^30^ By using the NMR‐based method described above, the percentage of weight loss of the enzyme‐treated GF‐PET, AP‐PET, and CP‐PET samples was determined as a function of the corresponding DP of the PET. The corresponding plot is shown in Figure 5c. With the highly amorphous GF‐PET sample, an apparent linear relationship was observed between the weight loss and the DP. A similar linear relationship was found with the AP‐PET and CP‐PET samples which showed a weight loss of up to 30%. In contrast, those samples incubated with the enzyme for longer times with a concomitant weight loss of more than 30% did not show a linear relationship suggesting that the observed reduction of the DP occurring at longer incubation times was not accompanied by a concomitant release of degradation products resulting in weight loss. A breakdown of the polymer chain resulting in weight loss of the material is characteristic of an exo‐type degradation mechanism releasing oligo‐ or monomers located at the ends of polymer chains.^31^ By contrast, an endo‐type degradation will result only in random chain scissions within the polymer and not leading to weight loss of the material.^31^ The latter has been previously suggested as the major mechanism of the enzymatic hydrolysis of PET^[12b]^ although a release of hydrolysis monomers^32^ or weight loss of bulky polymers[qv: 12c] indicating an exo‐type degradation mechanism were also observed. It is therefore hypothesized that the enzymatic hydrolysis of PET starts with an endo‐type chain scission after the adsorption and binding of the enzyme to an available ester bond randomly located in the polymer chain, independent of the initial polymer microstructures (Figure 5d). After this initial chain breakdown, the enzyme can further hydrolyze the neighboring ester bonds available in MAF along the polymer chain via an exo‐type chain scission, which results in the release of degradation products and weight loss of the bulky polymer. In contrast, due to the restricted chain mobility and conformation, neighboring ester bonds in RAF and crystalline PET are often not accessible to the enzyme which has to target far away located accessible ester bonds in a more time‐ and energy‐consuming manner, e.g., desorption and readsorption to the polymer surface. With this respect, the results shown in Figure 5c provide evidence that following the preferential degradation of the accessible MAF of PET by a combination of endo‐ and exo‐type chain scissions, TfCut2 further catalyzed only endo‐type chain scissions in the less hydrolysis‐susceptible RAF or crystalline microstructures of PET, however, at a much lower rate.

### 2.5 The Enzyme‐Treated PET Packaging Samples Showed a Changed Surface Morphology

**Figure 6:**
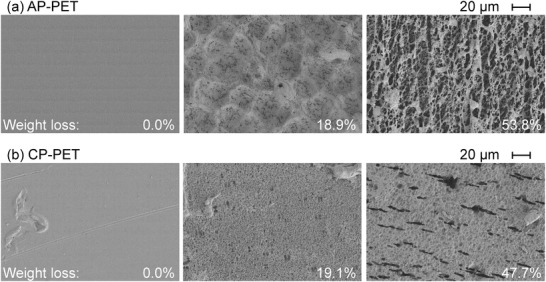
Surface modification of enzyme‐treated PET packaging samples analyzed by SEM. a) AP‐PET and b) CP‐PET chip surfaces at different extents of weight loss are shown.

Analysis of the AP‐ and CP‐PET samples by scanning electron microscopy (SEM) showed drastic changes in their surface morphology following an incubation with the enzyme (**Figure** **6**). The apparent cracks and uneven surfaces could reflect the preferential degradation of the amorphous microstructures in the materials with the more recalcitrant regions remaining. The observed sponge‐like surface structures may thereby represent crystalline microstructures not susceptible to enzymatic hydrolysis.

## 3 Conclusions

Using a recombinant bacterial polyester hydrolase TfCut2 expressed in *B. subtilis*, two different postconsumer PET packaging materials could be efficiently degraded as indicated by an apparent weight loss of up to 50% after incubation at 70 °C for 96 h. DSC analysis confirmed the presence of a MAF fraction of the PET which was more accessible to enzymatic hydrolysis and a RAF fraction which was more difficult to be hydrolyzed by the enzyme. The occurrence of RAF fractions was considered to be related to the presence of neighboring crystalline microstructures in the polymer as a result of a strain‐induced crystallization during the thermoforming manufacturing process of the packaging. During hydrolysis at 70 °C, a transition of MAF to RAF as a result of a physical aging process was observed which resulted in drastically decreased degradation rates during further incubation. An apparent relationship was observed between the amount of the crystalline microstructures in the PET samples and the corresponding degradation rates indicating a preferential hydrolysis of the MAF by the polyester hydrolase TfCut2. A physical aging process observed by incubation of the PET materials at 70 °C negatively influenced their enzymatic degradation. An NMR‐based approach was established to estimate the changes of the apparent degree of polymerization of the PET samples. The results suggested the presence of a combination of exo‐ and endo‐type chain scissions in the MAF whereas the RAF and the remaining crystalline microstructures of PET could be cleaved only in an endo‐type manner and at a much lower rate without concomitant weight losses.

Our findings suggest that thermostable polyester hydrolases with high hydrolysis efficiency against both amorphous and crystalline PET are required, not only aiming to the general low energy and time costs of the biorecycling process but also to reduce the conversion loss caused by the decreased polymer degradability as a result of the accompanying physical aging of PET during the enzymatic degradation.

## 4 Experimental Section

*Construction of Plasmids and Bacterial Strains: B. subtilis* strain RH 11496 was obtained by transformation with the plasmid pDATfCut2 as described previously.^33^ The high‐copy plasmid pDATfCut2 was constructed by amplifying the high‐copy number vector pDAM^34^ and the synthesized *tfcut2* gene, which encodes the *Thermobifida fusca* cutinase TfCut2,[qv: 15b] with optimized codon usage for *B. subtilis* introducing complementary sequences to the 3′‐ends with a melting temperature (*T*_m_) above 50 °C. AccuPrime Pfx DNA Polymerase (Life Technologies, Carlsbad, USA) was used for DNA amplification by PCR following manufacturer's instructions. Amplified products were purified using the Wizard SV gel purification kit (Promega, Madison, USA) and were fused by using the Gibson assembly kit (New England Biolabs, Ipswich, USA). The product was directly used to transform competent cells of the intermediate host *B. subtilis* DB104^35^ as described previously.^36^

*Recombinant Enzyme Production, Purification, and Characterization*: For the *B. subtilis* strain RH 11496 used in this study, cultivations of the seed cultures were carried out at 37 °C and 180 rpm in shaking flasks. The first preculture was prepared as an overnight culture of one colony from a lysogeny broth agar plate (10 g L^−1^ bacto‐tryptone, 5 g L^−1^ yeast extract, 10 g L^−1^ NaCl, and 10 g L^−1^ agar) with suitable antibiotics. The secondary inoculum was cultured from 5% (v/v) of the primary inoculum. For the fermentations carried out, the secondary seed cultures were used as an exponentially growing culture to inoculate 0.5 L fermenters with an inoculum size of 3.75% (v/v). The composition of the media used was as described previously.^37^ The cultivation was carried out at 37 °C and pH of 7.0 ± 0.2 which was automatically adjusted with NH_3_ and H_2_SO_4_ during the fermentation process of 42 h. The initial aeration and agitation rates were 0.5 vessel volume per minute (vvm) and 1200 rpm. Samples were taken over the entire fermentation process after a cultivation time of 2, 5, 16, 18, 20, 22, and 42 h. After a centrifugation for 15 min at 4500 rpm, the culture supernatant was used for the activity measurement and analyzed by sodium dodecyl sulfate polyacrylamide gel electrophoresis (SDS PAGE). The hydrolytic activity was assayed using *para*‐nitrolphenyl acetate as a substrate. One unit of hydrolytic activity was defined as the amount of enzyme required to produce 1 µmol of *p*‐nitrophenol per min at 20 °C and pH = 7.0. Purification of the recombinant TfCut2 was carried out by size exclusion chromatography (SEC) as described before.[qv: 15a]

Recombinant expression of TfCut2 in *E. coli* BL21(DE3) and the subsequent purification sequentially by immobilized metal ion affinity chromatography and SEC was performed as described before.[qv: 15b]

The thermal unfolding processes of purified enzymes were investigated by CD spectroscopy using a Jasco J‐715 spectropolarimeter (JASCO, Easton, USA). 10 × 10^−6^ m of purified enzymes were dissolved in 50 × 10^−3^ m sodium borate buffer (pH 8.0) in quartz cuvettes with a path length of 2 mm (Hellma, Jena, Germany). The thermal unfolding process was monitored by the ellipticity changes at 220 nm as a function of temperature from 55 to 95 °C in 0.5 °C steps.

*Enzymatic Hydrolysis of the PET Materials*: Amorphous PET film with a thickness of 250 µm was purchased from Goodfellow Ltd. (Bad Nauheim, Germany, product number ES301445; GF‐PET). Postconsumer PET packaging containers for fresh fruit and vegetables obtained from Agripack (Groupe Guillin, Ornas, France; AP‐PET) and Carton Pack (Carton Pack Srl, Rutigliano, Italy; CP‐PET) were also used. Fourier transform infrared spectroscopy (FTIR) measurements of all three PET types were performed in ATR (attenuated total reflection) mode with the FTIR spectrometer Vector 22 (Bruker Corporation, Billerica, MA, USA) and a diamond crystal (Golden Gate Specac, Orpington, UK). The spectra were obtained in the wavenumber range of 550–4000 cm^−1^ using a spectral resolution of 2 cm^−1^ (Figure S3, Supporting Information).

PET chips of 3 × 0.5 cm^2^ with an average weight of 38.9, 31.1, and 44.2 mg for AP‐, CP‐, and GF‐PET, respectively, were placed in a 2 mL reaction vial containing 1.8 mL of K_2_HPO_4_/Cl (1 m, pH = 8.0) and 1 nmol cm^−2^ of purified TfCut2. Degradation was performed by shaking the reaction vials on a thermoshaker TS1 (Biometra, Göttingen, Germany) at 70 °C and 1000 rpm. The reaction was stopped by cooling the samples on ice. PET chips were washed and dried as described previously and subjected to gravimetric determination of the weight loss.^38^

*DSC Analysis of the PET Materials*: Differential scanning calorimetry was performed using a DSC 8500 calorimeter (PerkinElmer, Waltham, USA) using ≈4–5 mg of a dry PET sample. A heating rate of 10 °C min^−1^ was applied for the temperature range from −20 to 300 °C. The glass transition temperature (*T*_g_), the cold crystallization temperature (*T*_cc_), and the melting point (*T*_m_) of various PET samples were obtained using the first heating scan. The initial fraction crystallinity *X*_0_ was calculated according to

(1) $$X_{0}=X_{\infty }\;+\frac{\Delta H_{\mathrm{cc}}}{\Delta H_{m}^{0}\left(T_{\mathrm{cc}}\right)}$$

(2) $$X_{\infty }=\frac{\Delta H_{m}}{\Delta H_{m}^{0}\left(T_{m}\right)}$$

(3) $$\Delta H_{m}^{0}\;\left(T_{\mathrm{cc}}\right)=\Delta H_{m}^{0}\;\left(T_{m}\right)-\Delta C_{p}\left(T_{m}-T_{\mathrm{cc}}\right)$$

as described before,^21^ where *X*_0_ is the initial crystallinity, *X*_∞_ is the complete crystallinity, Δ*H*_m_ is the melting enthalpy, and Δ*H*_cc_ is the cold crystallization enthalpy. $\Delta H_{m}^{0}(T_{m})$ is the melting enthalpy of pure crystalline PET at the melting temperature of 140 J g^−1^.^39^ $\Delta H_{m}^{0}(T_{\mathrm{cc}})$ is the melting enthalpy of pure crystalline PET at the temperature of cold crystallization, which can be calculated according to Equation (3). Δ*C*_p_ is the difference of the heat capacity of amorphous and crystalline PET of 0.17 J g^−1^ K^−1^.^21^

*NMR Analysis of the PET Materials*: The enzyme‐treated PET samples and untreated control samples were dissolved in hexafluoroisopropanol (HFIP, ≥ 99%, Merck KGaA, Darmstadt, Germany) and stored at room temperature over 120 h for a complete dissolution. The PET solutions with a final concentration of 7.14 µg µL^−1^ were used for the NMR analysis. 5 µL of each PET solution was pipetted into a 5 mm NMR tube and mixed with 80 µL chloroform‐*d* (CDCl_3_, 99.5% D, Cambridge Isotope Laboratories, Tewksbury, USA) containing 0.05% (v/v) tetramethylsilane (TMS) as an internal standard (δ^H^, 0.00 ppm). The solvent residual signal of CDCl_3_ calibrated on the TMS scale was used as a reference (δ^H^, 7.24 ppm).

All ^1^H solution NMR experiments were performed with a Bruker DRX‐600 NB spectrometer equipped with a 5 mm TXI probe (Rheinstetten, Germany) at 290.3 ± 0.1 K. An optimized ^1^H 90° pulse of 6.0 µs and a spectral width of 12.02 kHz was employed. The data were recorded with 256 or 1024 scans and a recycle delay of 15 s. A twofold zero‐filled to 128k points and an exponential line broadening of 0.2 Hz was applied to data processing. The baseline was corrected manually afterward. Processed data were analyzed with MestReNova 12.0.0 software (Mestrelab Research, Santiago de Compostela, Spain). For determination of the ^1^H signal of the β CH_2_ protons for the hydroxyl end group, its ^13^C satellite at the low‐field side was not involved in the calculation due to their heavily overlapping with the strong main signal of the CH proton in HFIP (a septet centered at 4.41 ppm).

*SEM Analysis of the PET Materials*: The surface morphology of PET samples were determined by SEM (Ultra 55 SEM, Carl Zeiss Ltd., Göttingen, Germany) with samples coated with a thin (30 nm) chromium film using a Z400 sputter system (Leybold, Hanau, Germany).

## Conflict of Interest

The authors declare no conflict of interest.

## Supporting information

Supplementary

## References

[1] PlasticsEurope , Plastics ‐ The Facts 2017, PlasticsEurope, Brussels, Belgium 2018.

[2] World Economic Forum , The New Plastics Economy: Rethinking the future of plastics, Cologny, Switzerland 2016.

[3] a) C. M. Rochman , M. A. Browne , B. S. Halpern , B. T. Hentschel , E. Hoh , H. K. Karapanagioti , L. M. Rios‐Mendoza , H. Takada , S. Teh , R. C. Thompson , Nature 2013, 494, 169; 23407523 10.1038/494169a

[4] J. Baeyens , A. Brems , R. Dewil , Int. J. Sustainable Eng. 2010, 3, 232.

[5] F. Awaja , D. Pavel , Eur. Polym. J. 2005, 41, 1453.

[6] R. Geyer , J. R. Jambeck , K. L. Law , Sci. Adv. 2017, 3, e1700782. 28776036 10.1126/sciadv.1700782 PMC5517107

[7] a) R.‐J. Mueller , Process Biochem. 2006, 41, 2124;

[8] a) R.‐J. Mueller , I. Kleeberg , W. D. Deckwer , J. Biotechnol. 2001, 86, 87; 11245897 10.1016/s0168-1656(00)00407-7

[9] R. Wei , T. Oeser , W. Zimmermann , Adv. Appl. Microbiol. 2014, 89, 267. 25131405 10.1016/B978-0-12-800259-9.00007-X

[10] S. Yoshida , K. Hiraga , T. Takehana , I. Taniguchi , H. Yamaji , Y. Maeda , K. Toyohara , K. Miyamoto , Y. Kimura , K. Oda , Science 2016, 351, 1196. 26965627 10.1126/science.aad6359

[11] Ã. M. Ronkvist , W. Xie , W. Lu , R. A. Gross , Macromolecules 2009, 42, 5128.

[12] a) M. A. Vertommen , V. A. Nierstrasz , M. Veer , M. M. Warmoeskerken , J. Biotechnol. 2005, 120, 376; 16115695 10.1016/j.jbiotec.2005.06.015

[13] F. Quartinello , S. Vajnhandl , J. Volmajer Valh , T. J. Farmer , B. Vončina , A. Lobnik , E. Herrero Acero , A. Pellis , G. M. Guebitz , Microb. Biotechnol. 2017, 10, 1376. 28574165 10.1111/1751-7915.12734 PMC5658601

[14] Y. Yang , M. Malten , A. Grote , D. Jahn , W. D. Deckwer , Biotechnol. Bioeng. 2007, 96, 780. 16948171 10.1002/bit.21167

[15] a) J. Then , R. Wei , T. Oeser , M. Barth , M. R. Belisário‐Ferrari , J. Schmidt , W. Zimmermann , Biotechnol. J. 2015, 10, 592; 25545638 10.1002/biot.201400620

[16] L. Westers , H. Westers , W. J. Quax , Biochim. Biophys. Acta, Mol. Cell Res. 2004, 1694, 299. 10.1016/j.bbamcr.2004.02.011 15546673

[17] M. Barth , R. Wei , T. Oeser , J. Then , J. Schmidt , F. Wohlgemuth , W. Zimmermann , J. Membr. Sci. 2015, 494, 182.

[18] D. Carta , G. Cao , C. D'Angeli , Environ. Sci. Pollut. Res. 2003, 10, 390. 10.1065/espr2001.12.104.8 14699998

[19] a) B. Hegemann , A. Kech , U. Göschel , K. Belina , P. Eyerer , J. Macromol. Sci., Part B: Phys. 2002, 41, 647;

[20] a) J. Zhao , J. Wang , C. Li , Q. Fan , Macromolecules 2002, 35, 3097;

[21] R. M. R. Wellen , E. Canedo , M. S. Rabello , J. Mater. Res. 2011, 26, 1107.

[22] a) N. M. Alves , J. F. Mano , E. Balaguer , J. M. Meseguer Duenas , J. L. Gomez Ribelles , Polymer 2002, 43, 4111;

[23] a) J. Menczel , B. Wunderlich , J. Polym. Sci., Polym. Phys. Ed. 1980, 18, 1433;

[24] R.‐J. Mueller , H. Schrader , J. Profe , K. Dresler , W.‐D. Deckwer , Macromol. Rapid Commun. 2005, 26, 1400.

[25] a) J. M. Hutchinson , Prog. Polym. Sci. 1995, 20, 703;

[26] D. P. Dowling , J. Tynan , P. Ward , A. M. Hynes , J. Cullen , G. Byrne , Int. J. Adhes. Adhes. 2012, 35, 1.

[27] a) A. Launay , F. Thominette , J. Verdu , Polym. Degrad. Stab. 1994, 46, 319;

[28] a) A. M. Kenwright , S. K. Peace , R. W. Richards , A. Bunn , W. A. MacDonald , Polymer 1999, 40, 2035;

[29] a) I. Seganov , J. M. Schultz , S. Fakirov , J. Appl. Polym. Sci. 1986, 32, 3371;

[30] F. Kawai , M. Oda , T. Tamashiro , T. Waku , N. Tanaka , M. Yamamoto , H. Mizushima , T. Miyakawa , M. Tanokura , Appl. Microbiol. Biotechnol. 2014, 98, 10053. 24929560 10.1007/s00253-014-5860-y

[31] X. Han , J. Pan , Acta Biomater. 2011, 7, 538. 20832507 10.1016/j.actbio.2010.09.005

[32] A. Eberl , S. Heumann , T. Bruckner , R. Araujo , A. Cavaco‐Paulo , F. Kaufmann , W. Kroutil , G. M. Guebitz , J. Biotechnol. 2009, 143, 207. 19616594 10.1016/j.jbiotec.2009.07.008

[33] S. Chang , S. N. Cohen , Mol. Gen. Genet. 1979, 168, 111. 107388 10.1007/BF00267940

[34] T. N. Ploss , E. Reilman , C. G. Monteferrante , E. L. Denham , S. Piersma , A. Lingner , J. Vehmaanperä , P. Lorenz , J. M. van Dijl , Microb. Cell Fact. 2016, 15, 57. 27026185 10.1186/s12934-016-0455-1 PMC4812647

[35] F. Kawamura , R. H. Doi , J. Bacteriol. 1984, 160, 442. 6434524 10.1128/jb.160.1.442-444.1984 PMC214740

[36] C. Anagnostopoulos , J. Spizizen , J. Bacteriol. 1961, 81, 741. 16561900 10.1128/jb.81.5.741-746.1961 PMC279084

[37] C. Cadot , T. Ploss , R. Schwerdtfeger , B. Winter , (EU Patent Germany), *EP2145006 B1*, 2011.

[38] R. Wei , T. Oeser , J. Schmidt , R. Meier , M. Barth , J. Then , W. Zimmermann , Biotechnol. Bioeng. 2016, 113, 1658. 26804057 10.1002/bit.25941

[39] A. Mehta , U. Gaur , B. Wunderlich , J. Polym. Sci., Polym. Phys. Ed. 1978, 16, 289.

