# Supplementary material for: Biocatalytic Degradation Efficiency of Postconsumer Polyethylene Terephthalate Packaging Determined by Their Polymer Microstructures
Source: Adv Sci (Weinh). 2019 May 20;6(14):1900491. doi: 10.1002/advs.201900491 (PMC6662049; doi:10.1002/advs.201900491)
Supplement: Supplementary file 1 — Supplementary [file ADVS-6-1900491-s001.pdf]

Copyright WILEY-VCH Verlag GmbH & Co. KGaA, 69469 Weinheim, Germany, 2019.

ADVANCED SCIENCE
Open Access

Supporting Information

for *Adv. Sci.,* DOI: 10.1002/advs.201900491

Biocatalytic Degradation Efficiency of Postconsumer Polyethylene Terephthalate Packaging Determined by Their Polymer Microstructures

Ren Wei, Daniel Breite, Chen Song, Daniel Gräsing, Tina Ploss, Patrick Hille, Ruth Schwerdtfeger, Jörg Matysik, Agnes Schulze, and Wolfgang Zimmermann**

Copyright WILEY-VCH Verlag GmbH & Co. KGaA, 69469 Weinheim, Germany, 2018.

Supporting Information

Biocatalytic Degradation Efficiency of Post-Consumer Polyethylene Terephthalate Packaging Determined by Their Polymer Microstructures

Ren Wei, Daniel Breite, Chen Song, Daniel Gräsing, Tina Ploss, Patrick Hille, Ruth Schwerdtfeger, Jörg Matysik, Agnes Schulze, Wolfgang Zimmermann

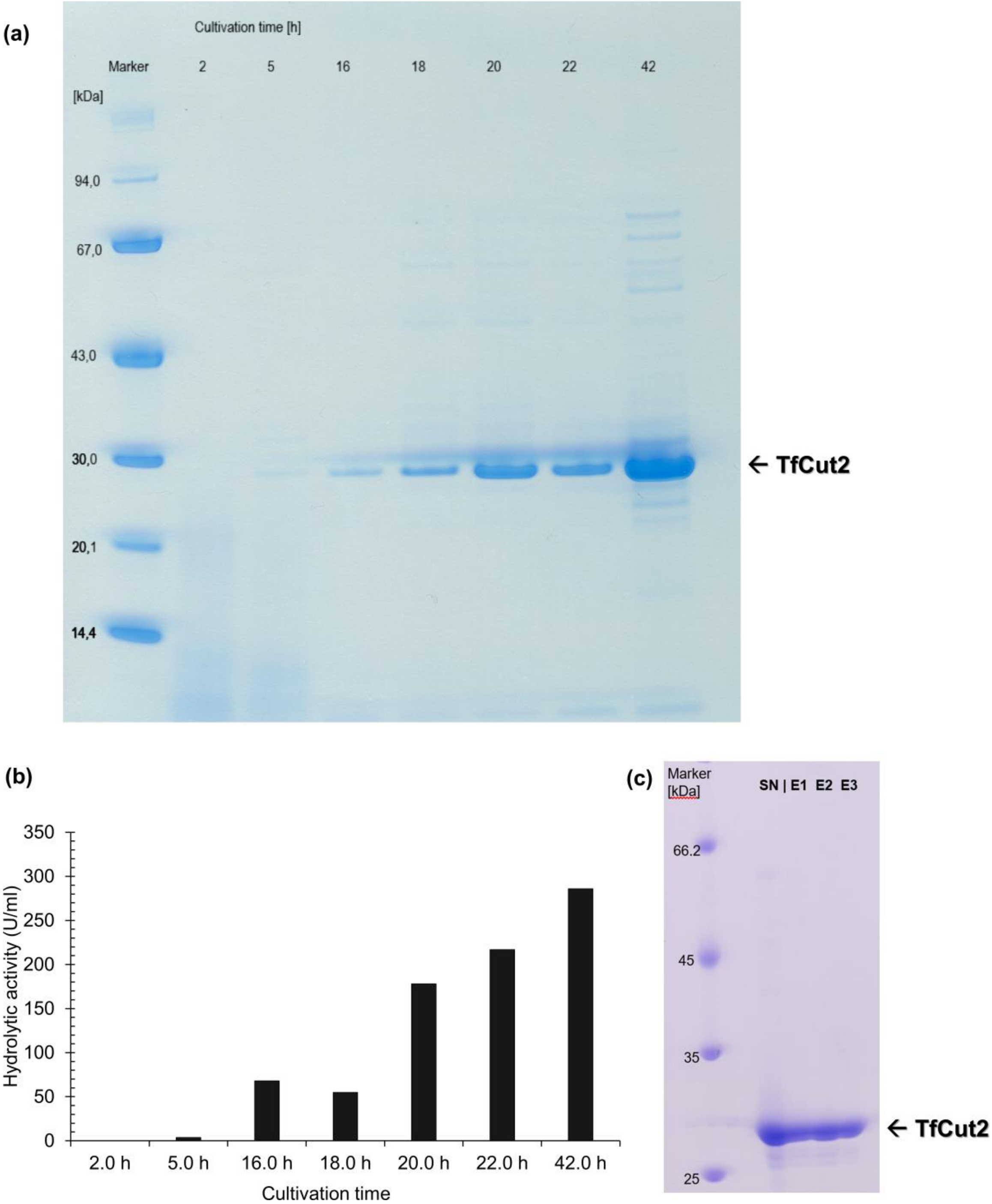

Figure S1. (a) SDS PAGE analysis of 6.5 µl of cell-free culture supernatant removed at indicated time points of *B. subtilis* cultivation for TfCut2 production. (b) Activity measurement of secreted TfCut2 over the entire fermentation process of *B. subtilis* RH 11496. PET esterase activity per ml culture is defined as one unit activity producing 1

µmol of *p*-nitrophenol by hydrolyzing the substrate *p*-nitrophenyl acetate in 1 min at 20°C at a pH of 7.0. (c) SDS PAGE of TfCut2 containing culture supernatant indicates the yield of highly pure TfCut2 by one step SEC purification. SN: culture supernatant, E1-3: protein elution fractions 1 to 3. 10 µL sample was loaded in each lane.

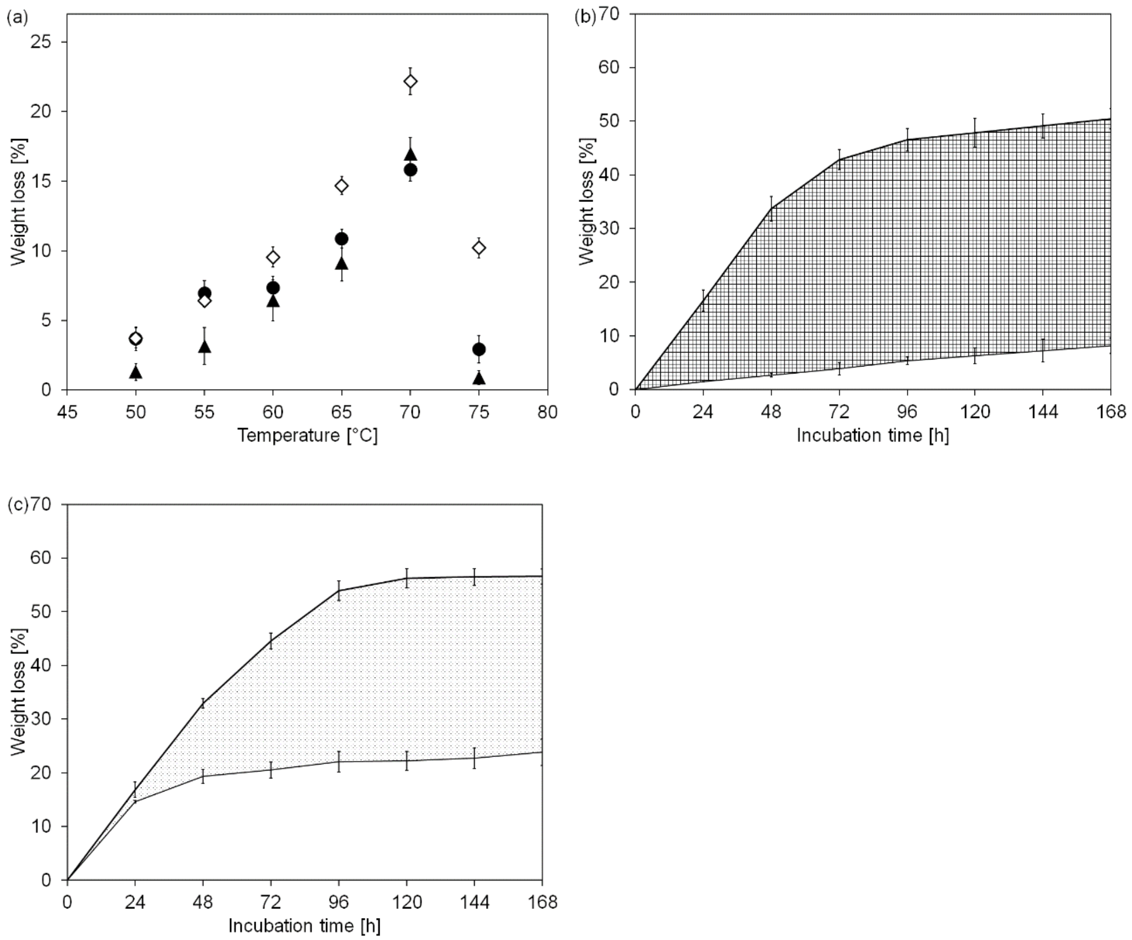

Figure S2. (a) Averaged percentage weight losses of GF-PET (diamonds), AP-PET (filled triangles) and CP-PET (filled circles) chips (0.5×3 cm^2) after an incubation time of 24 h at different temperatures by TfCut2. Data of post-consumer PET samples were collected using the most hydrolysis-susceptible parts. Time courses of the weight loss of AP-PET (b) and CP-PET chips (c) sampled from different parts of the packages

hydrolyzed by TfCut2 for 168 h indicate a wide range of degradability of the post-consumer PET. Error bars indicate the standard deviations obtained from at least triplicate experiments. Shaded areas indicate the minimum and maximum weight losses of more than 16 chips sampled from different parts of the PET packages.

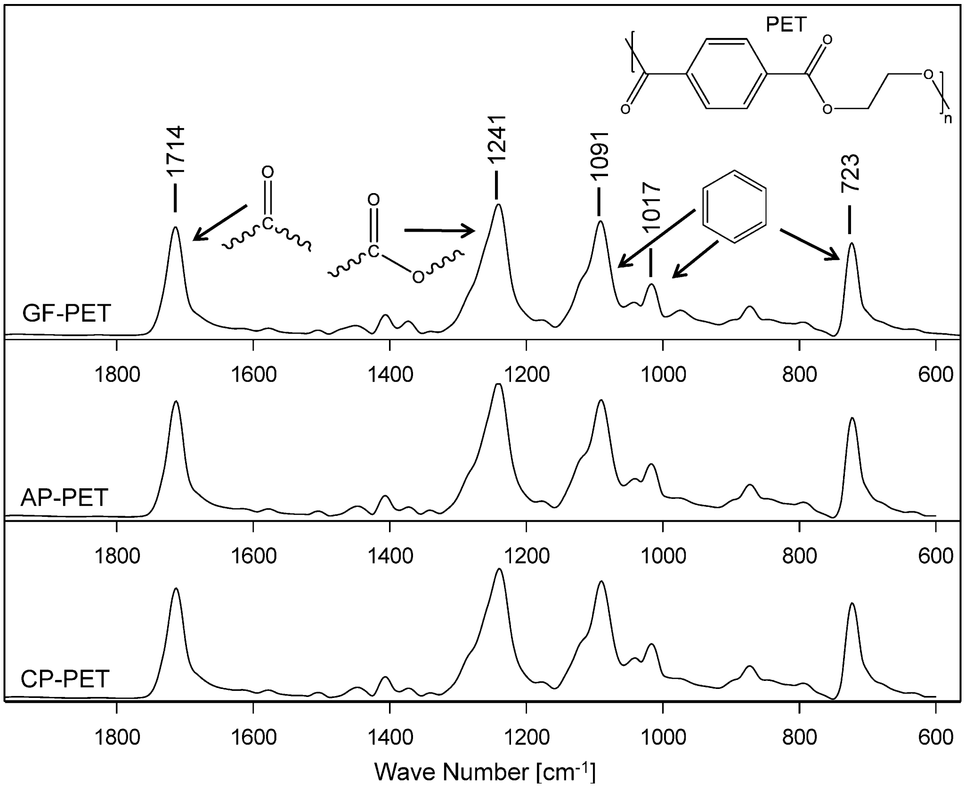

Figure S3. ATR-FTIR of (a) GF-PET, (b) AP-PET, and (c) CP-PET. ATR mode was used to detect differences of the top layer of the samples. The spectra show the characteristic signals of PET and the absence of other compounds in the samples.
